# Hospitalized children's experience of a Fairy Garden in Northern Thailand

**DOI:** 10.1002/nop2.482

**Published:** 2020-03-19

**Authors:** Pamela van der Riet, Chaweewan Jitsacorn, Peter Thursby

**Affiliations:** ^1^ School of Nursing and Midwifery Faculty of Health and Medicine The University of Newcastle (UoN) Newcastle NSW Australia; ^2^ Boromarajonani College of Nursing, Ministry of Public Health Lampang Thailand; ^3^ Educational Consultant Newcastle Australia

**Keywords:** Hospitalized children, narrative inquiry, therapeutic landscapes

## Abstract

**Aim:**

To explore through draw, observation and talk hospitalized children's experience of a Fairy Garden in Northern Thailand.

**Design:**

A participatory visual arts method of draw, observations and talk along with Clandinin's narrative inquiry framework of three‐dimensional space (sociality, temporality and place).

**Methods:**

This study was conducted in a paediatric ward in a large tertiary hospital in Northern Thailand over 12 months (July 2017–2018). A purposive sample of 17 hospitalized children aged between 4 and 13 years participated in drawings and face‐to‐face interviews. The study adhered to the COREQ checklist for qualitative research (see Supplementary File S1).

**Results:**

The analysis resulted in five interrelated threads: (1) representations of the illness experience; (2) imagination and fantasy; (3) connection to place, family and home; (4) moments of social representations of play; and (5) happiness and enjoyment.

## 1 INTRODUCTION

This study explored the experiences of sick children hospitalized in a Northern Thailand hospital who had access to and were able to take part in recreational activities in a natural and play area titled a Fairy Garden (FG) located adjacent to the two children's wards in the hospital. An increasing body of research indicates the value of green space such as gardens and recreational facilities to the overall health and well‐being of hospitalized children (McCormick, 2017).

How children respond to the facilities of an FG and the impact of such a facility on their well‐being as hospital patients can contribute to our knowledge in better catering for children in hospital. These green spaces have sometimes been referred to as healing environments (Ananth, 2008), and an evaluation of healing gardens in a hospital setting for paediatric cancer patients indicates potential for this “restorative environment” to overcome the pressures of hospitalization for children (Sherman, Varni, Ulrich, & Malcarne, 2005). Healing environments can be described in terms of both nonphysical and physical to facilitate the healing process impacting through psychological and spiritual dimension of health (Abbas & Ghazali, 2012). Findings from the study by Sherman et al. (2005) concluded that while adults prefer to sit, socialize and walk about the garden, sick children will actively engage with facilities in the garden.

The focus of several studies has been on hearing the voice of children as they reveal their views of their experiences while in hospital (Brady, 2009; Corsano et al., 2012, 2015; Wilson, Megel, Enenbach, & Carlson, 2010). Wilson et al.’s (2010) study looked at the emotions of children through story telling of their hospitalized experience. Brady’s (2009) study used draw and talk and addressed hospitalized children's views on what makes a good nurse. Corsano's et al. (2015) study looked at the emotional reaction to hospitalization, and Corsano's et al. (2012) study looked at the relationship with nurses and doctors.

The purpose of the study was not to investigate children's views of what makes a good nurse and their relationships with healthcare professionals but, instead, to explore the experiences of children hospitalized in a Northern Thailand hospital who were able to engage in recreational activities in a natural garden and play area titled a Fairy Garden (FG). The FG is a facility with gardens of colourful plants and flowers, short walks through the gardens, play equipment, a cubby house, bridge, gazebo, concrete blue whale, ceramic animals and seating for quiet times with parents or carers (van der Riet, Jitsacorn, Junlapeeya, Thursby, & Thursby, 2014). All hospitalized children had access to the FG throughout the day providing they did not have an infectious illness. The Fairy Garden was easily accessed from both wards so children could engage freely in activities during the day providing they were not receiving any scheduled treatments such as chemotherapy or blood transfusions. Parents, grandparents and nurses would accompany both small and any unwell children while some children entered the garden unaccompanied. Nursing staff were always nearby to monitor activity in the FG.

## 2 BACKGROUND (LITERATURE)

The value of nature in urban design through the inclusion of green spaces to benefit human psychological and physical well‐being (Hoyle, Hitchmough, & Jorgensen, 2017) has also been advocated in the healthcare industry through the provision of natural environments and outdoor spaces being incorporated into hospital design for the potential healing benefits afforded to patients (Dijkstra, Pieterse, & Pruyn, 2006; Geary, 2003; Horowitz, 2008; Huisman, Morales, Hoof, & Kort, 2012; Ingulli & Lindbloom, 2013; Morrison, 2011; Pasha, 2013; van der Riet, 2014; Walker, 2012; Zborowsky & Kreitzer, 2009). Abbas and Ghazali (2012) recommended design features to be included in newly built paediatric wards to include natural and therapeutic gardens, easy access to gardens, play areas and garden activities as best practice in supporting better outcomes for young patients. Whitehouse et al. (2001) examined levels of satisfaction of a garden environment in a designed hospital for sick children and found that patients, family and hospital staff rated the facility highly in providing garden seating, plants and flowers, play equipment and spaces that took sick children away from the pressures of clinical care. Pasha and Shepley (2013) emphasized the importance of design characteristics that would allow hospitalized children to engage in more active behaviours through provision of spaces for play activity, sculptures, gardens, pathways and inclusion of shading for warmer climates. In examining design trends in hospitals in terms of facilitating healing, Ghazali and Abbas (2012) indicated that as well as the physical environment users’ satisfaction focused on additional activities to engage patients, art, music and pet therapy.

More recent studies had also explored the family member's experience of the FG (van der Riet, Jitsacorn, Junlapeeya, Thursby, & Thursby, 2017a) and nursing students’ experiences (van der Riet, Jitsacorn, Junlapeeya, & Thursby, 2017b). However, there is limited literature on sick children's views of natural and recreational environments in hospital design. What is particularly beneficial for further research is the examination of sick children's experience of the garden environment where there are facilities to walk, receive visual stimulation, play and interact with others.

The aim of the study was to explore through draw, observation and talk hospitalized children's experience of a Fairy Garden in Northern Thailand. Our research question was as follows: What was the children's experience of a Fairy Garden in a hospital environment?

## 3 DESIGN

A narrative inquiry approach comprising of participant observation and participatory visual arts‐based activity was used. Narrative inquiry (NI) is the study of experience understood narratively (Clandinin, Caine, Lessard, & Huber, 2016), that is through a representation of events (Clandinin, 2013). Experience in NI is a narratively created phenomena with NI viewed as both methodology and phenomenon (Clandinin, 2013). As our study involved narrative inquiry, the children's experience of the FG was the phenomena we were studying.

## 4 METHOD

### 4.1 Data collection

The study took place in a paediatric unit in a Northern Thailand regional hospital. The paediatric unit consisted of two medical and surgical wards with 70 inpatient beds.

### 4.2 Participants

Participants were eligible for inclusion if they were children aged four years and over and deemed well enough to participate in the research activities. We selected the age of four as Irwin and Johnson (2005) have previously advised that in qualitative research with children as young as four years there can be discerning data.

Eligible participants and parents were approached by the nurses on the wards. Each of the 17 children who were approached provided assent to participate in the drawing activities. However, one child declined to draw. This child participated in the interview, so we still included him in the study.

### 4.3 Procedure

A drawing station was established in the precinct of the paediatric ward with table and chairs. Drawing material was available for each child consisting of white paper and a variety of coloured pencils. The children chose which coloured pencils they would use in the drawing and were asked to draw their experiences of the FG. After their drawing, each child was interviewed about their drawings (Table 1).

**TABLE 1:** Participant characteristics

Name of participant	Age and gender	Diagnosis	Length of stay in hospital
Tilly	4 years. Female	Neuroblastoma	2 days, however repeated admissions to the ward
Jill	4 years. Female	Right hernia repair	2 days. Previous admission to the ward for left hernia repair
Pippy	8 years. Female	Ruptured appendix Appendectomy	4 days
Minny	5 years. Female	Recto vesicular fistula. For colostomy the next day	2 days. Repeated admissions
Kim	11 years. Female	Headaches, nausea, vomiting, blurred vision. For neurological investigation and CT scan	3 days
Elsa 1	7 years. Female	Productive cough and fever for investigation	5 days
Grape	13 years. Female	Type I diabetes	4 days
Elsa 2	5 years. Female	Infection in her leg	1 day
Vier	7 years. Male	Appendectomy Epilepsy	4 days
Namcha	12 years. Female	Type 1 diabetes	6 days
Pisue meaning Butterfly	10 years. Female	Juvenile arthritis	3 days
Messey	11 years. Male	Epilepsy	4 days
Banana	9 years. Female	Anxiety and associated disorder admitted for abdominal pain	7 days and numerous admissions
Mickey	6 years. Male	Appendectomy	4 days
Kitty	9 years., Female	Abdominal pain	3 days
Ninja	10 years. Male	Appendectomy	4 days
Diamond	13 years. Female	Glioblastoma	7 days. Numerous admissions, craniotomy 12 months ago

Initially, any uncertainty as to the task was managed by friendly talk to place the children at ease and was conducted in Thai by the Thai researcher. Questions of the children included the following: had they done drawing before, did they like to draw and were they happy to make a drawing that told a story about their experience of the FG? Simple language was used to minimize any misunderstandings and to check if the children were comfortable in participating. The emphasis was on the children telling their story through drawing so that they could present what was important to them and then to talk about their drawing to clarify their experience.

The interviews consisted of the Thai translator (who was part of the research team) and the principal researcher (who was not Thai) were conducted through the Thai translator. Interviews were translated simultaneously at the time of the interview and audio recorded, and each researcher made a running record that became a journal of the process. Recordings and researcher's notes were also later checked for accuracy. The principal researcher had considerable experience in conducting interviews in Thailand with Thai research colleagues. An introductory explanation of the study and the semi‐structured questions were prepared in English and then translated to Thai by the Thai researcher. While the children were drawing, the researchers made observation notes using a simple grid, noting how long the children drew, expressions on their faces, colours chosen and used. We also noted if they thought about what they were doing before they started drawing or if they went straight into the activity. The amount of time the children drew varied from 15 min to one hour. These observations in our grid and interviews assisted us with our analysis.

### 4.4 Participant characteristics

Most of the children were from rural areas and regional provinces in Northern Thailand. Parents were mostly farmers or small business owners.

### 4.5 Data analysis

Analysing the data was an iterative process. Our data included the children's drawings, our observations of them doing the drawings and our conversations with them after they completed their drawings. Selected examples of the children's drawings were included in support of the analysis along with observations and postdrawing discussions with children to support our findings.

Consisted of three stages:

1. In analysing the data, we drew up a table with 4 columns that included details of the participants, content of the drawing and what the participants said about their drawing and lastly our suggested threads. In this table, we also highlighted words that fitted with potential threads. Furthermore, we listened carefully to the translations of audio voice recordings from both the children and ourselves.
2. A coding schema adapted from Boles and Winsor’s (2019) study of children with cancer involved looking at the colours, objects and human figures. We also looked for physical markers of illness, cognitive expressions, social expressions and emotional expressions (Boles & Winsor, 2019).
3. Further to analysing content of the drawings, observations (noted in our grid), conversations with the children along with our notes we used the three metaphorical dimensional space involving temporality sociality and place. (Clandinin, 2013) These three dimensions of inquiry are central to the conceptual framework of narrative inquiry (Clandinin, 2013) and helped shape the design of the study (Clandinin et al., 2016). Clandinin (2013) points out the importance of researchers attending in temporal ways as it takes the researcher to the past, present and future of the person's experience. As narrative inquiry researchers, we also needed to attend to the dimension of sociality involving personal conditions (hopes and dreams) and social conditions (cultural, institutional and familial) (Clandinin, 2013). We also needed to attend to the third dimension, that is place, with a focus on the physical concrete and the topographical boundaries of place where the inquiry and events took place (Clandinin et al., 2016). In the context of the study, place is the FG in a hospital setting between two paediatric wards.

### 4.6 Ethics approval

This study was approved by the first author's University Ethics Committee and local approvals from the hospital. Informed written consent was provided by parents or grandparents who were the main carers. The study adhered to the Consolidated Criteria for Reporting Qualitative Research (Appendix S1) (Tong, Sainsbury, & Craig, 2007). Family members gave written consent freely, and children gave their verbal assent. Each participant was asked to select their own special name for the study (pseudonym). Names were chosen from a favourite movie (mostly female participants), a sports person (male participants) and for some a favourite alternative name to their own.

## 5 RESULTS

In analysing the children's drawings and their interviews, five interrelated threads were identified and interwoven in this article: representations of the illness experience; imagination and fantasy; connections to place, familial‐family and home; moments of social representations of play; and happiness and enjoyment. Consistent with the work of Clandinin (2013), we have replaced themes with threads. These threads are what has resonated across our conversations, observations and in the drawings of the children. Most of these threads are interwoven and relational and difficult to isolate as just single threads.

The dimensions of place (mostly the FG and home), time (past, present and future) and sociality (desire and feelings) are central in all the threads of this study.

### 5.1 Threads

#### 5.1.1 Representations of the illness experience

**FIGURE 1:**
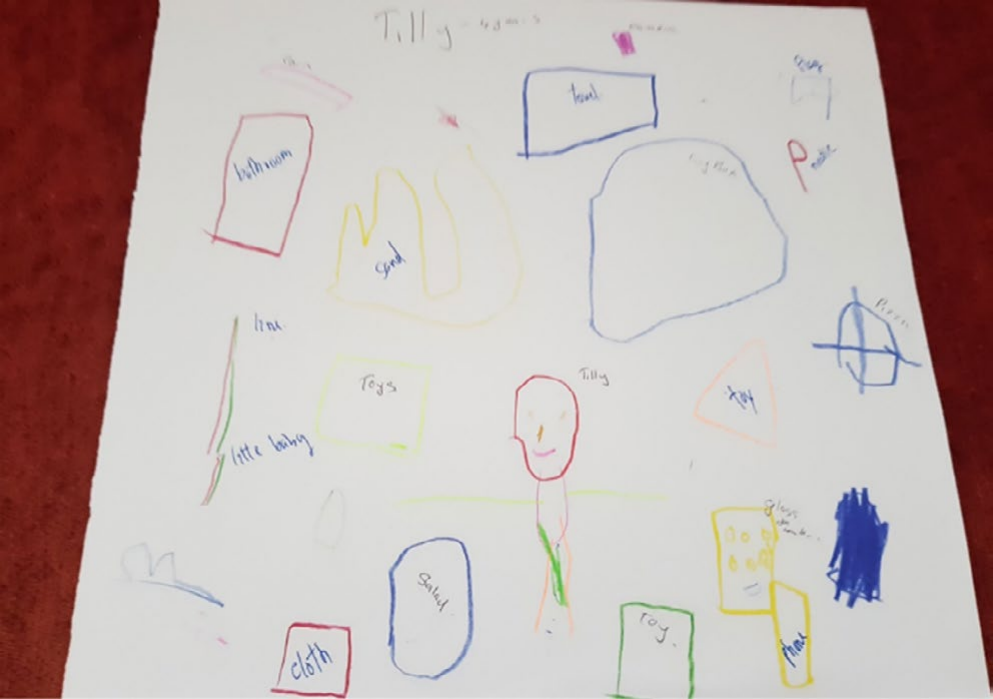
Tilly's drawing

Several drawings presented imagery that fell into more than one thread. The illness experience was presented in several drawings. Tilly (Figure 1) drew a series of simple shapes and lines that she associated with particular objects. The illness experience in Tilly's drawing relates to what she missed. The cancer treatment for her neuroblastoma had involved chemotherapy, which would cause her to react to food and limit her enjoyment of eating. The representation of herself with no hair is another feature of her treatment although within her age group figurative schemas tend not to include many details. Items of food feature in her drawing and conversation with us:

I want to eat pizza, corn and salad when I am in the FG.

We wondered if this was because she was unable at times to eat due to the side effects of nausea and vomiting from her chemotherapy. Along with favourite foods, there are named images of a bathroom, a towel and drinking cups that may represent her personal care due to illness. We noted that the range of imagery in her drawing presented the dimension of temporality (past, present and future) with an emphasis on eating food in the FG.

For Diamond, a 13‐year‐old participant newly diagnosed with a glioblastoma, the exercise of drawing helped her adjust to being in hospital and to her aching head. In her interview, Diamond reported:

I love art and doing this drawing has helped take away my headache.

The activity allowed her to concentrate on something she liked doing. Her drawing (Figure 6) focused on specific objects and things in the FG that she would have actively engaged with during hospitalization. We were not actually aiming in this study to find that drawing would be therapeutic; however, in this instance drawing gave Diamond a voice and may well have had the potential to help her adjust to her illness and in personally focusing on a positive experience.

For Namcha, 12 years of age with diabetes type 1 the FG as place became a distractor where she could forget her illness and the clinical boundaries of the paediatric ward. This is confirmed in her conversation:

When I sit on the swing in the FG I get a feeling that I am not being bothered about anything and the movement backwards and forwards helps me not feel bored.

However, we noted that she did not draw herself in her drawing, only those objects of specific interest such as the swings, wishing well, garden walk and cubby house. Namcha also told us:

I love to sit near the wishing well and I sit there for 30 min.

In retrospect, we did wonder if she did make a wish (future desire) at the wishing well in the FG.

#### 5.1.2 Imagination and fantasy

**FIGURE 2:**
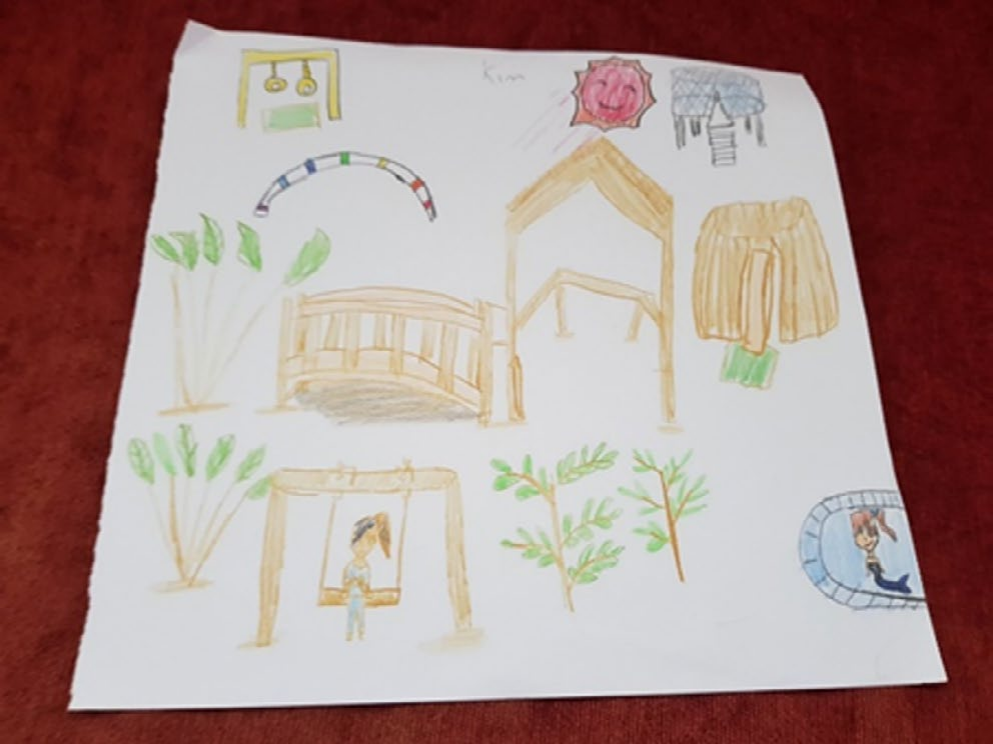
Kim's drawing

Imagination and fantasy were apparent as a thread in several children's drawings. Through this thread, the dimensions of temporality, sociality and place are evident. Sociality is represented in the expression of feelings and desire to be in the present. Kim reported that she had not yet taken part in any activities in the garden, yet she placed herself in the drawing (see Figure 2) sitting on the swing. She is shown wearing hospital attire (blue clothing representing herself in the present moment) and with an expressive attractive hairstyle (a desire to look nice). A series of individual items such as plants and gazebo from the FG are drawn together with additional images of items Kim imagines could exist in the garden such as a mermaid in a pool, a smiling sun, a trampoline and gymnastic rings hanging on a frame.

**FIGURE 3:**
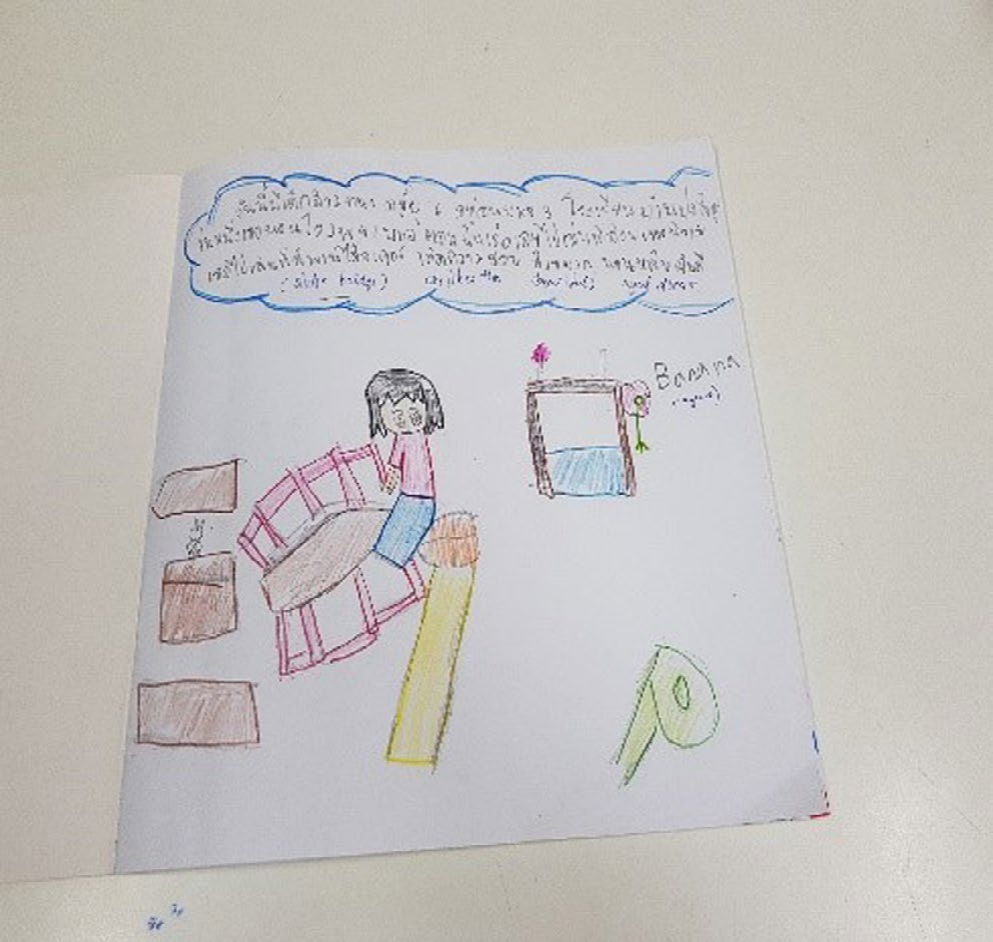
Banana's drawing

Figure 3 presents the drawing created by Banana, who had multiple admissions to the unit presenting with unresolved symptoms of headache and abdominal pain and a history of anxiety and dissociative disorder. Her drawing shows a small selection of objects from the garden (bridge, slide, swing and wishing well) that she liked. She is seen sitting on the slide, one of her favourite activities in the garden.

Above the drawing Banana has written a story about a friend, a six‐year‐old girl, who once went to hospital and played in the garden. We did wonder if her drawing represented an escape from her reality and was perhaps linked to her diagnosis of dissociative disorder. Her own feelings about the garden are like those of her friend, they both liked the garden, especially the slide, and if they could not sleep, they went into the garden to play on the slide, the bridge and the swing. Banana's written story stated that playing in the garden gave them beautiful feelings, made her and her friend happy and sleepy and have sweet dreams.

Banana concentrated on her drawing and during the interview was relaxed and happy. We did wonder about the therapeutic nature of this, if drawing and the telling of her story through the drawing made her feel relaxed and in control of events surrounding her. Attending to the dimensional spaces of time, place and relationship in NI, Banana focused strongly on her time and place in the garden and her imagined relationship with a little friend who enjoyed the same things she did.

**FIGURE 4:**
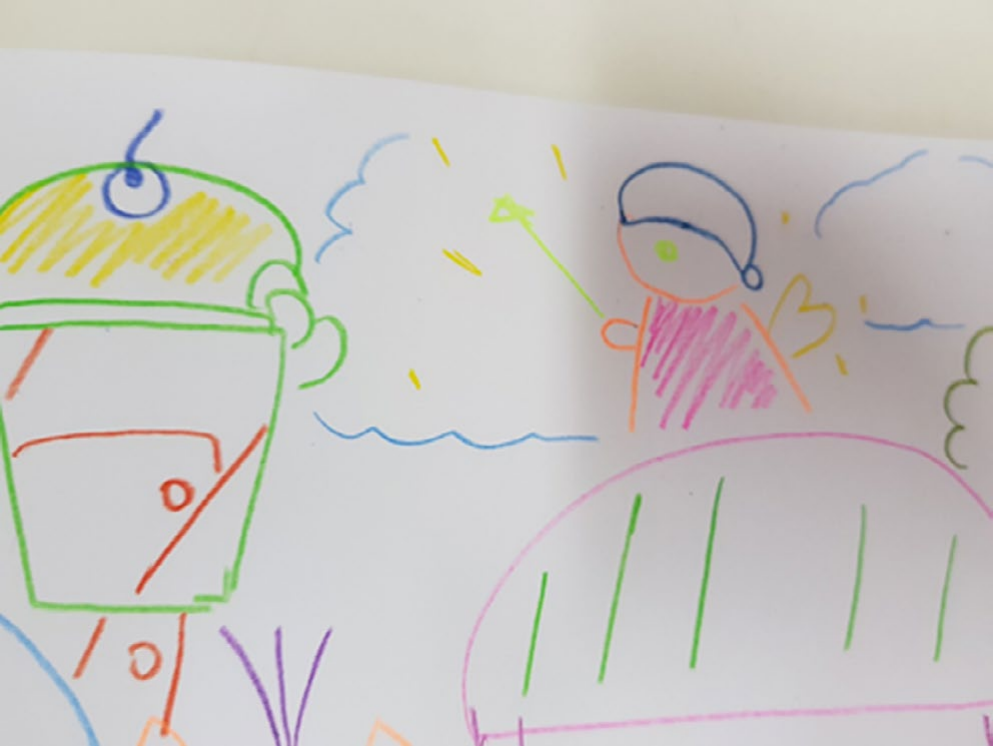
Grape's drawing

**FIGURE 5:**
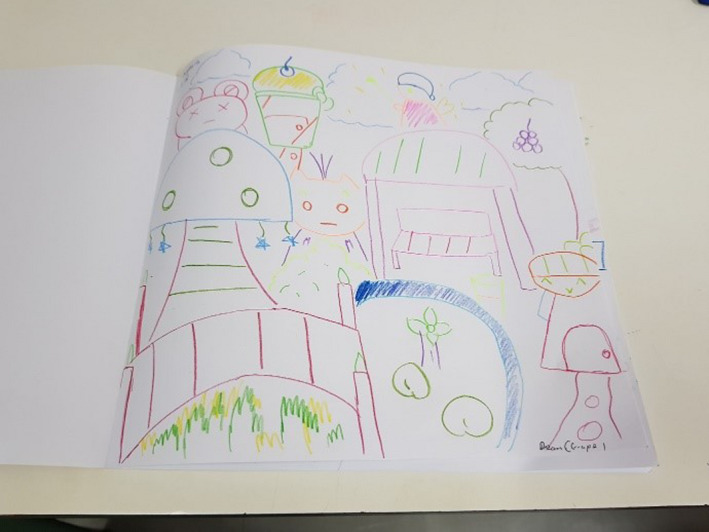
Grape's drawing

Food was very central in Grape's drawing (Figures 4 and 5) and in her conversation. She has drawn an angel at the top of the page and pointed out:

This angel has magic and can get rid of my illness so that I can eat whatever I like. Before I became unwell with diabetes, I could do everything and eat everything that I liked. I could be like a normal teenage girl. Now I have lost my lovely lifestyle of eating sweet food.

In her drawing, there is magic in her wishes and desires and this is further confirmed in her interview. Grape reported that she did not play on the items in the garden as most were too small for her and also she felt too tired and preferred to walk in the garden or sit on one of the seats. When asked how she felt while in the garden, she said:

I feel like a child again!

There was a degree of fantasy in Diamond's drawing that was interwoven with a desire for play.

**FIGURE 6:**
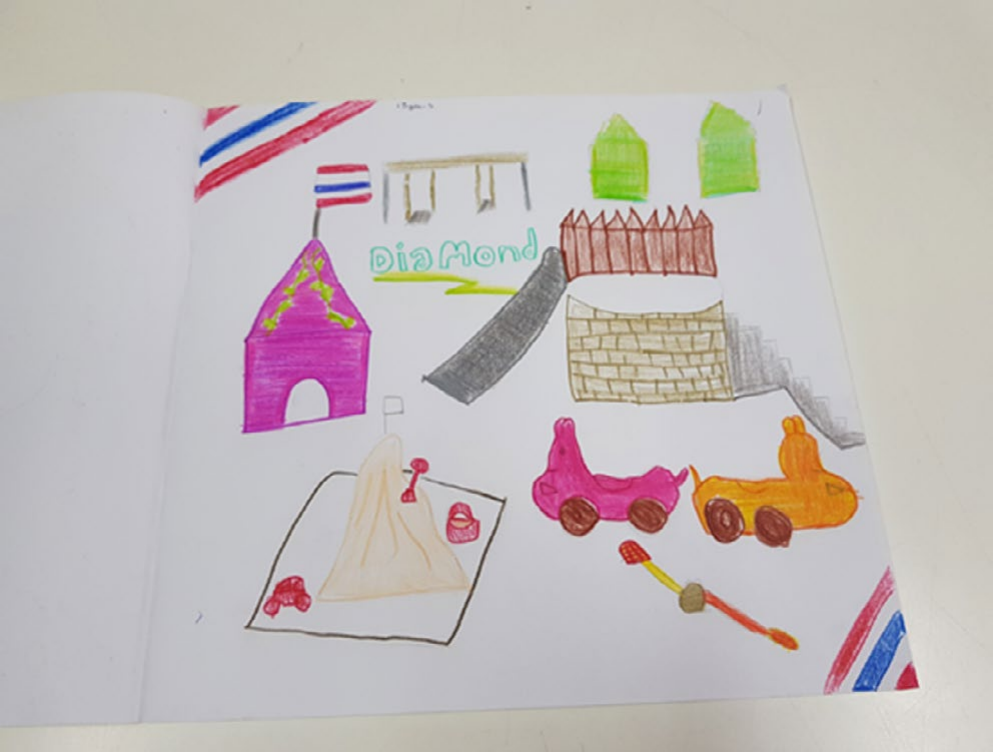
Diamond's drawing

Diamond tells us that she loves art, and we note that she has drawn a sandcastle (Figure 6) in her drawing. She recounted:

I would like to have a sandcastle and to be able to play in the sand.

We wondered if this was a previous experience, she enjoyed and wanted to return to at that time or in the future. However, in Northern Thailand there are no beaches. She saw the FG as a place for physical activity, imaginative play and an opportunity to make and build a sandcastle. The Thai flag was placed in a top and bottom corner and on the cubby house. The garden, contained within a rectangular shape, has been added in an imaginative way to include a sandcastle, digging tools, bucket and toy castle. The colours create a bright environment through her choice of a wide range of colours to emphasize each item. In her representation of the garden, it suggests a conscious desire to tell a story about the FG as a place of imaginative activity for her. The metaphorical dimensions of time (past, present, future), sociality (an inner desire) and place are captured in this drawing.

#### 5.1.3 Connections to place; familial‐family and home

This thread seems to be intertwined with imagination and fantasy of the previous thread, and there are strong representations of the dimensions: sociality, place and temporality.

**FIGURE 7:**
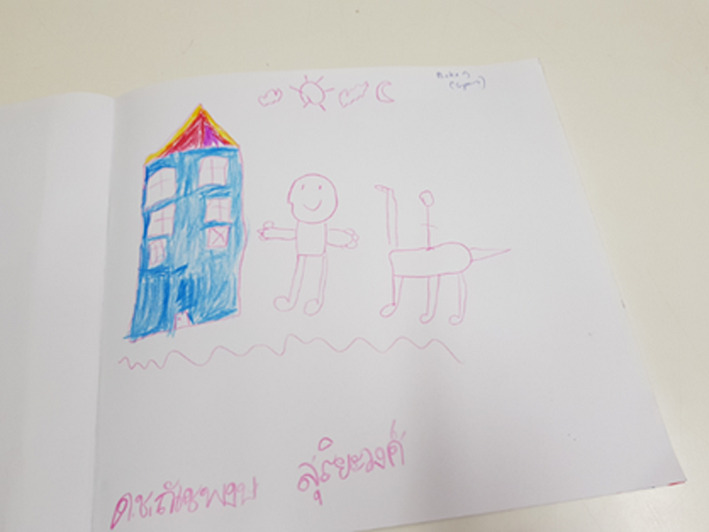
Mickey's drawing

For several of the children, there is a very close desire for connection to place and family, place being home. For Mickey (Figure 7), there were lots of fantasy images and memory about family (sociality) in his drawing. Mickey has reimagined the gazebo from the FG and presented a house of three stories, with a gable roof and windows. The figure representing Mickey stands next to the gazebo, and an animal, the rocking horse, from the FG is shown next to the figure with a rider. At the top of the page, there are images symbolizing clouds, the sun and the moon. While drawing, Mickey links his experience of the FG to his family, particularly his grandfather who was his main carer. His grandfather is central in his story and here reflected in his statement that:

I miss my grandfather and every time I go into the FG I think of him and playing together.

The dimension of sociality is evident in Mickey's drawing as he spoke of bringing his family into the experience of the FG and connection to family eased that he was in an unfamiliar place, other than home. Identification of place through play becomes an overarching sense of geographical location. We asked ourselves was this a desire for home as Mickey is an inpatient? He used the colour blue a lot in his drawing, and he told us that his grandfather liked blue.

There is also a strong sense of cultural patriotism representing the dimension of sociality in several of the children's drawings by including the Thai flag. Importantly, in NI experience is continuous, relational and social (Clandinin, 2013). This is evident in this thread along with the temporality of the past represented in Mickey's drawing, along with place in the present and future for his grandfather to be with him in the FG.

#### 5.1.4 Social representation of play

**FIGURE 8:**
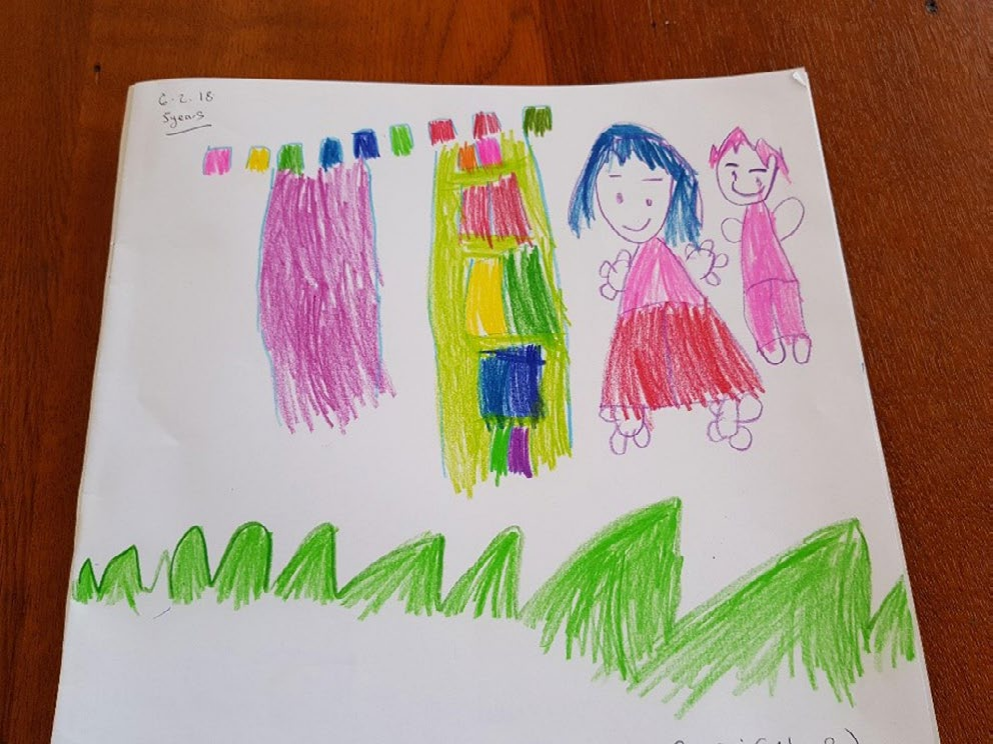
Elsa 2's drawing

Interestingly, there were only two participants who drew other children in the garden with them. Both Minny and Elsa 2 added second figures in acknowledgement of the social character of the FG. Threads of social representation and happiness and enjoyment intermingle in presenting the figures together as each figure is colourfully drawn with smiling faces. Elsa 2 (Figure 8) drew a little boy next to her as they played in the FG and they wear pink pyjamas provided by the hospital.

Several drawings by the children symbolized play and engagement with social activities characteristic of daily activities, signifying the dimension of sociality. For example, Tilly drew (see Figure 1) a yellow sand pit, a bathroom and toy box and there is a little baby. She clearly liked the FG and told us that she played in the garden three times a day. Her drawing crosses threads to consciously express the illness experience as well.

The FG represented for Tilly a place of play and fun with items relevant to her at her age We also note from our observations that Tilly really engaged and immersed herself in the FG and she was very eager to show things in the garden that she liked, that is the swing, swinging bridge and slide, all play equipment involving movement. After the interview, Tilly was very active running around in the FG, moving backwards and forwards, spending time socializing at the table with the other children who were doing their drawings and being interviewed. Although this was a little disruptive, we did not have the heart to send her away, so just let her be. We were aware in our research that with such a vulnerable participant we needed to be flexible in our approach to data collection.

In Minny's drawing, there were a series of items from the garden including cubby house, swing and small ceramic animals. Minny has drawn herself in the garden and next to her is another child. Each is represented with bright colours, expressive faces and hair in patterns to represent a boy and a girl.

#### 5.1.5 Happiness and enjoyment

**FIGURE 9:**
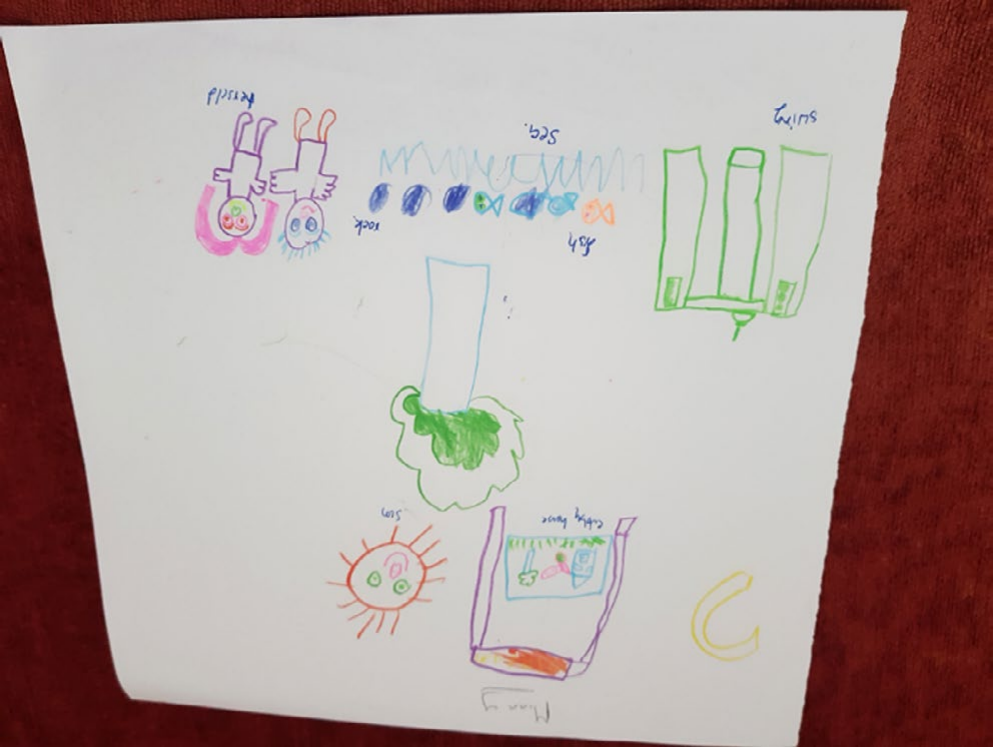
Minny's drawing

The thread of happiness and enjoyment is probably the strongest of all the threads with acknowledgement to the dimension of sociality and place. All the children told us that the garden made them feel happy and joyful and that it was beautiful place to come and play as there were nice things to see and do. Many of the drawings with figures presented happy, smiling faces, and none of the drawings indicated negative emotional expressions such as frowns, tears or downturned mouths. In Kim's drawing (Figure 2), there was a lovely smiling sun above the gazebo, and Minny (Figure 9) had a smiling sun looking over the garden. Minny stated that she liked drawing herself in the garden and that she liked to sit on the whale, the swing and the slide. Even Tilly, despite her life‐limiting illness of a neuroblastoma, was still full of energy and appeared from our observations happy engaging in the FG.

Happiness is evident in the following comments from participants and is interwoven with the previous thread of play, subsequently signifying the dimension of sociality:

The FG gives me beautiful feeling and I am happy playing in the garden with the other children (Banana).

I love to make drawings and being in the garden. It makes me happy and I want to play in the garden all of the time. (Elsa 2)

Kitty made a colourful drawing of the things she liked and told us she played in the FG every day. She enjoyed the swing and the slide and has drawn herself waiting to take a turn on the swing. The swing occupies two‐thirds of the upper part of the page and has a pattern of hearts and diamonds on the ropes. When we asked her how she felt when she visited the garden, she said:

I feel enjoyment and fresh, compared to when I am inside of the hospital. In the FG I play three times a day in the morning, afternoon and in the evening.

**FIGURE 10:**
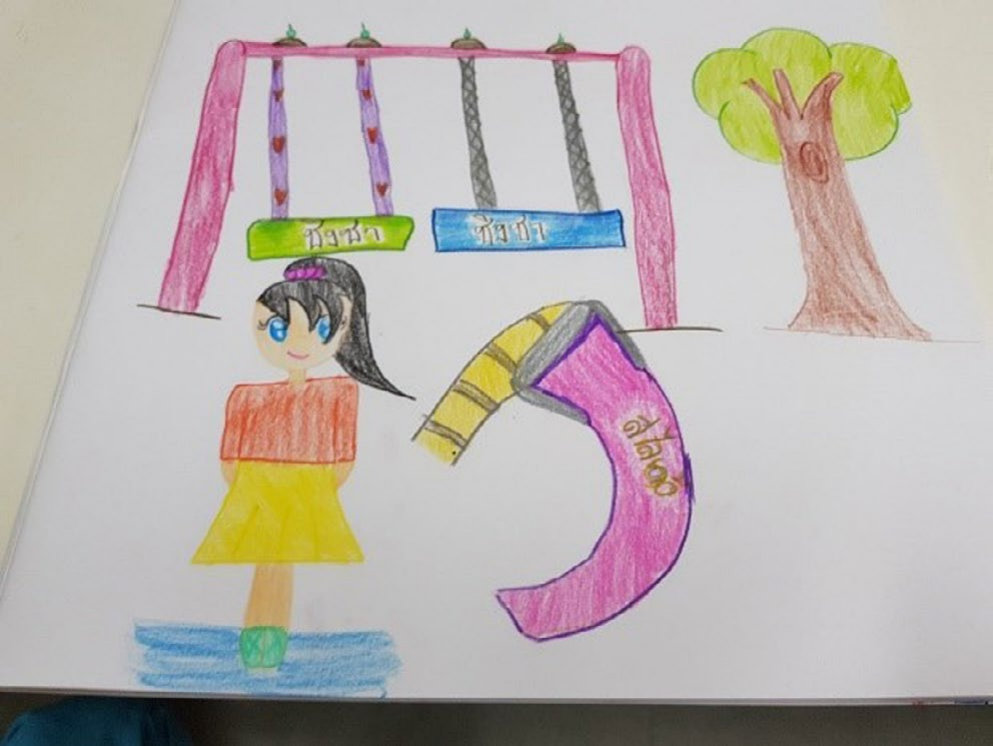
Kitty's drawing

There is a strong personal link here to the place of the FG in her experience and involvement with the garden and a sense of happiness to have such a place for relief and distraction from the clinical environment (Figure 10).

**FIGURE 11:**
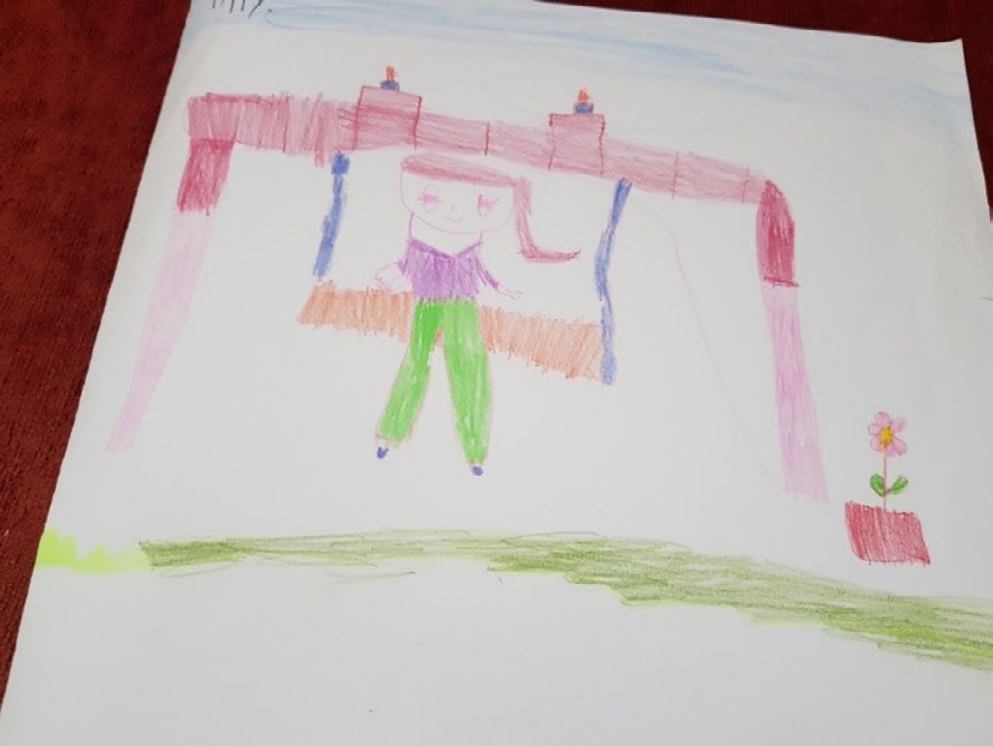
Pippy's drawing

During the interview, Pippy had an intravenous cannula in her right hand. She still wanted to draw even though we offered her not to do a drawing if this created discomfort for her. We note that she did not draw herself with a cannula in her hand. In her drawing, Pippy sitting on the swing had a happy face with decorative eyes embellished by prominent eye lashes and attractive hair style. The structure of the swing has a decorative pattern of rectangular shapes, and the figure and objects have been carefully coloured (Figure 11).

## 6 DISCUSSION

Findings that have emerged in this study suggest that the children perceived the FG through a lens of illness experience, imagination, fantasy and connection to place and family. It brought enjoyment and happiness for all of them. In the data, there are links to the past, present and future and this came in through their drawings and conversations (mostly about feelings) with us. The discussion now turns to what can be learnt from hospitalized children's experience of a FG and application of what we have learned for future practice. The FG was a means in closing the spatial gap between the biomedical model of illness and a therapeutic environment of play and fun. There is a need to rethink models of care for children that includes elements of humanized care that focuses on environment spaces that encourage social play and fun. Wilson's et al. (2010) study explored children's stories about hospitalization and identified that playing with others helped improve their hospitalized experience. The importance of play, especially outdoors in nature, has been the topic of pilot studies conducted by primary care paediatricians who have given prescriptions to children to spend more time outdoors in nature in play activities (Christiana, Battista, James, & Bergman, 2017).

McCormick’s (2017) systematic literature review involving the impact of green space on children's mental well‐being identified that it improved stress and well‐being of children and recommended healing gardens in hospital settings to enhance health by promoting outdoor play. Ozcan (2006) argues that paediatric patients are more sensitive than adult patients to the environment and, therefore, the impact of healing is more significant when there is engagement with healing environments in the hospital.

Best practice in paediatric wards supports the creation of healing environments that include opportunities for play and accessibility to outdoor therapeutic gardens (Abbas & Ghazali, 2012). The thread of happiness and imagination is consistent with other studies on the FG with the nurses and also student nurses reporting that they observed happiness and there was a sense of imagination and play (van der Riet et al., 2014; Riet, Jitsacorn, Junlapeeya, Thursby, & Thursby, 2017a; Riet, Jitsacorn, Junlapeeya, Thursby, & Thursby, 2017b). In previous studies, play in the garden was seen as a distractor from illness (van der Riet et al., 2014) and this was consistent with what we found. In particular, the FG offered an escape from the boredom of the clinical environment. The children in this study all reported in the interview the FG made them feel happy and gave them joy.

Attention to relationship building was important in this research for several reasons. Firstly, it is important as we were researching with a vulnerable population and several of the children were doubly vulnerable as they had a terminal diagnosis (glioblastoma and neuroblastoma). We were conscious that the children with cancer are a doubly vulnerable cohort due to their very young age and life‐limiting and threatening illness (Boles, 2018). Boles and Winsor (2019) have emphasized the importance of building rapport with children and parents. Furthermore, Huang et al. (2014) emphasized the importance of developing child‐friendly strategies in building rapport with children to ensure a trustful and equitable relationship exists. Unbeknown to the children participating in the study, the researchers gathered together a variety of small gifts for the children following completion of their interview. Examples of gifts included a small tea set, comic books, dinosaurs and drawing material. Each child chooses a gift. As researchers, this was a way of honouring and thanking them for their participation. We praised the children on their drawings.

### 6.1 Limitations and strengths

A limitation of this study could well be that that we really did not look in detail at the development levels of the children's drawings, that is the mental schemas that show representation of images. We have not reported in detail any gender differences in the children's drawings, and this could be seen as another limitation of the study. Our strengths, however, are that we were flexible in our approach to our research and acknowledge data collection methods need to be flexible in researching with children (Boyles, 2018). For example, we were accommodating in enabling each child to take time when drawing. We allowed Tilly to freely come and go during the draw and talk processes, and we were encouraging in enabling the children's voices to be heard.

Another important strength was our method of using drawing to communicate the children's lived experience of the FG. Drawings provided possibilities of how children perceive and communicate their experiences of their world (Carter & Ford, 2013). In drawing, children are able to engage and express their views and imaginations (Driessnack, 2006). This point is further explained from The United Nations Convention on the Rights of the Child 1989/1990 UNCRC in article 12–13 which points out that children have a right to express their experience through forms of representation such as text, spoken words and visual methods (Khoja, 2016). There are also studies that have concluded that drawings give children access to a voice in telling their stories that they might otherwise have difficulty in presenting (Brady, 2009; Pipe, Salmon, & Priestley, 2002; Weinle, 2002; Wesson & Salmon, 2001).

It has also been asserted that drawing may promote children's agency (Greene & Hogan, 2005; Mannion, 2007). The opportunity for young children to present their stories places the emphasis on the child as agent of their experience and have their experience and be honoured. There is also recent literature that points out the importance of having a child's perspective rather than taking the child's perspective (Bryan, Bluebond‐ Langner, Kelly, Kumpunen, & Oulton, 2018; Coyne & Harder, 2011; Nilsson et al., 2015). In the co‐construction process of research, Lim and Lim (2013, p. 65) remind us that “both adult and child are equal players and the resulting dialogical process plays a major role in the constitution of the phenomena.”

Boles (2018) argues for a culturally appropriate and child‐centred approach to research that captures both verbal and non‐verbal data. More recent work by Boles & Windsor (2019) support data collection that is child‐centred and directed by the child. Furthermore, in our study we chose draw, observation and talk as a method to capture both verbal and non‐verbal data. In essence, the method of drawing a child's experience can be seen as data directed by the child.

Another strength was that we engaged in a high level of reflexivity. For example, straight after the interviews with the children both researchers met to discuss and clarify any tensions or concerns. We were very mindful in our approach to our observations, the interviews and analysis of the data. Having raised the issue of reflexivity, we now address this as a means of ensuring trustworthiness and rigour in our qualitative study.

### 6.2 Rigour and trustworthiness

We would argue that in establishing trustworthiness and rigour in our study our strong focus on reflexivity ensured our research demonstrated rigour and trustworthiness. Reflexivity has certainly become a critical topic in qualitative research (Shaw, 2016) and an elusive term (van der Riet, 2012) that requires the researcher to operate on multiple levels (Etherington, 2004) through self‐awareness and ensuring there is methodological cohesion. For example, ensuring the research question and aim match the methodology (van der Riet, 2012). In our study, the research aim involved experiences of a FG and this was clearly cognizant with a NI approach that focuses on the phenomena of experience. We were mindful that the stories that we told through the children's drawing were both our stories from the participants and the researchers. This is consistent with narrative inquiry as it is relational methodology.

Trustworthiness of the data is also linked to credibility, dependability, confirmability and transferability (Guba & Lincoln, 1994). In relation to dependability and credibility, we kept a very careful audit trail in keeping journal notes, meeting with each other straight after the interview to discuss any differences and confirm our understandings. This procedure was important, especially as the main author was not from the Thai culture. Credibility is achieved through triangulation of the data, and we achieved this with the transcripts, drawings, field notes and our reflective journals. Our triangulation of data assisted in ensuring data saturation.

Generalizability has become a controversial topic (Carminati, 2018). We would argue that generalizability is at odds with narrative inquiry methodology and fits more with a positivistic paradigm of quantitative research. Narrative inquiry researchers attend to participants’ stories as they are composed over time and in relation to people and situations at various different places and social settings so there is less likelihood of generalizations and certainties (Clandinin, 2013) in NI studies such as ours.

## 7 CONCLUSION

Through the physical representation of drawings and conversations with the children, we saw in this study a different world, one where there is an escape from the medical representation and boundaries of hospitalization. The FG brought a community/play environment that counters the biomedical and clinical environment. We would argue that healing environments and green space such as the FG have had a positive experience for the children in this study and provide a more humanized approach to the care of hospitalized children. Globally, a top priority for healthcare services now in the area of paediatric services is to build more natural landscapes for hospitalized children to engage in play, lessen stress and escape the boredom of clinical unwelcoming spaces.

## 8 RELEVANCE TO CLINICAL PRACTICE

Creating therapeutic environments for sick children benefits their hospital experience and provides more humanized care in helping to provide an escape from the clinical environment and assuage the stress of medical procedures.

## CONFLICT OF INTEREST

We have no conflicts of interest to declare.

## AUTHOR CONTRIBUTIONS

PV, PT: Data design. PV, CJ: Data collection. PV, PT, CJ: Data analysis.

## Supporting information

Supplementary Material
